# Cost-effectiveness analysis of different screening and diagnostic strategies for sexually transmitted infections and bacterial vaginosis in women attending primary health care facilities in Cape Town

**DOI:** 10.3389/fpubh.2023.1048091

**Published:** 2023-03-02

**Authors:** Elise Smith, Lindi Masson, Jo-Ann S. Passmore, Edina Sinanovic

**Affiliations:** ^1^Health Economics Unit, School of Public Health and Family Medicine, Faculty of Health Sciences, University of Cape Town, Cape Town, South Africa; ^2^Division of Medical Virology, Institute of Infectious Diseases and Molecular Medicine, Faculty of Health Sciences, University of Cape Town, Cape Town, South Africa; ^3^Life Sciences Discipline, Burnet Institute, Melbourne, VIC, Australia; ^4^National Health Laboratory Service, Cape Town, South Africa

**Keywords:** HIV prevention, point-of-care testing, cost-effectiveness, bacterial vaginosis, sexually transmitted infections

## Abstract

**Background:**

Genital inflammation associated with sexually transmitted infections (STIs) and bacterial vaginosis (BV) is considered a key driver in the HIV epidemic. A new rapid point-of-care test (POC) that detects genital inflammation in women—Genital InFlammation Test (GIFT)—was recently developed by researchers at the University of Cape Town. The objective of this study was to establish the cost-effectiveness of this novel intervention relative to other relevant screening and diagnostic strategies for the management of STIs and BV in women seeking care in the public health sector in South Africa.

**Methods:**

A decision analysis model was developed for five different screening and diagnostic strategies for women incorporating syndromic management, screening with GIFT and using etiological diagnosis. A decision tree was constructed using Microsoft Excel Office 365, and cost and effectiveness parameters were obtained from published literature and market prices. The model incorporated all clinic-level and treatment costs associated with diagnosing and treating a single episode of disease. The effectiveness of each approach was proxied by its sensitivity. One-way and threshold sensitivity analyses were conducted to test key uncertainties and assumptions in the model.

**Results:**

Screening with GIFT, and following with antibiotic treatment according to syndromic management guidelines for GIFT-positive cases, was the most cost-effective strategy with an incremental cost-effectiveness ratio (ICER) of USD 11.08 per women diagnosed with an STI(s) and/or BV and provided treatment. This strategy resulted in lower rates of overtreatment compared to syndromic management, but higher rates compared to etiological diagnosis using nucleic acid amplification tests and microscopy. However, following a GIFT positive test with etiological diagnosis prior to treatment did not increase the effectiveness, but dramatically increased the cost.

**Conclusion:**

Screening with GIFT and treating positive cases according to syndromic management guidelines is the most cost-effective strategy for the management of STIs and BV. GIFT has a potential to significantly improve the management of STIs and BV in women by identifying asymptomatic women and reducing their risk of HIV infection. This analysis presents a first step in establishing the cost-effectiveness of these interventions and paves the way for further research to develop optimal context-specific implementation strategies.

## Background

Sexually transmitted infections (STIs) and bacterial vaginosis (BV) represent a significant challenge to global public health. In 2012 the World Health Organization (WHO) reported that annually, around 499 million new cases of the four most common curable STIs (*Chlamydia trachomatis, Neisseria gonorrhoeae, Trichomonas vaginalis* and Syphilis infections) occurred globally (1). Recent WHO estimates confirmed that the majority of these occur in the developing world and that an annual incidence of 92 million new infections is accounted for in the African region alone (2). Some studies have also estimated that up to 55% of women in Sub-Saharan Africa have BV, which is considered a microbial dysbiosis rather than an infection (3). STIs and BV are regarded as one of the leading causes of disability adjusted life years lost in women (4). If left untreated, STIs and BV can lead to various serious sexual and reproductive complications, including increased risk of HIV acquisition and transmission (4–7).

South Africa houses one of the largest burdens of curable STIs in the world, with high prevalence in women who are at high risk of HIV (8–10). Given that the public health sector serves around 84% of the population, the vast majority of this burden rests on the South African National Department of Health (11). In the South African public health system, as in many low and middle-income countries (LMICs), most STIs and BV are managed syndromically, rather than with resource-intensive etiological diagnosis (12). Using the syndromic management approach developed by the WHO and modified to fit the South African context, syndromes of a specific STI or BV are identified according to pre-specified groups of signs and symptoms. Patients are then provided with treatment that will address the majority, or the most serious, of organisms typically associated with the identified syndrome (see Appendix A.1) (12, 13).

Given that the majority of these infections and BV are asymptomatic in women [estimates from South African studies range from 75 to 88% (14–17)], most cases are missed under the current standard of care since these women experience no symptoms and thus seek no care (17, 18). The accuracy of syndromic management is further undermined by the fact that syndromes and signs of the different STIs and BV overlap (19). Research done in South Africa suggests that nurses in the public sector have limited knowledge regarding appropriate treatment for the various syndromes (20, 21). Studies also report low specificity of this approach, resulting in overdiagnosis and overtreatment and thus excessive use of antibiotics. The latter has implications for the development of drug-resistant strains of various bacteria [like *N. gonorrhoeae* (22)] which is a growing concern, globally (13, 19).

Globally and locally, there is thus a need to move away from syndromic management and toward more effective management strategies of STIs and BV. More specifically, there is an urgent need to improve STI management for women in resource-constrained settings (18, 23). Research also indicates that improvements in STI management can significantly improve HIV prevention, especially in settings where both HIV and curable STIs are prevalent (17, 24, 25).

The gold standard for diagnosing STIs are laboratory-based nucleic acid amplification tests (NAATs), and microscopic identification through Nugent scoring for BV (26–28). These methods are, however, expensive and resource-intensive, making it unfeasible in resource-constrained settings (18). Furthermore, it does not allow for immediate results and requires that patients return to health care facilities to obtain results and receive treatment. More transmissions may take place during the waiting period. In addition, the proportion of women who will return to the clinic is often very low [as low as 11% in the African context (29, 30)], consequently affecting treatment.

With the WHO's ambitious goal of a 90% reduction in the incidence of STIs and zero new infections by 2030, the improved detection and treatment of asymptomatic STI and BV cases form a key part of the organization's STI prevention and control strategies. In this context, the WHO has prioritized the development of relevant point-of-care (POC) tests (1, 31). In South Africa, under the new National Sexually Transmitted Infections Strategy, and as part of the Western Cape's Provincial Strategy Plan, zero new HIV and STI infections also form part of the long-term vision for public health in South Africa. This is within the context of the overarching framework for health which strives toward achieving universal coverage for all, and that prioritizes the health of vulnerable populations (32, 33).

In reaction to growing concerns about the affordability of etiological diagnosis of STIs and the performance of the syndromic management approach in women, more rapid and less expensive NAATs have been developed. Cepheid's GeneXpert CT/NG test is an example of a combined chlamydia and gonorrhea POC NAAT from the USA that is commercially available in South Africa. A similar assay has also been developed detecting trichomoniasis; GeneXpert TV. These tests perform comparatively well to laboratory based NAATs and are ideally performed in on-site laboratories associated with reproductive healthcare clinics, using the GeneXpert system, and can present results in roughly 90 min. However, they are still relatively expensive in an LMIC context, and are thus not widely available on-site at most healthcare facilities (17, 34, 35).

Recently, researchers at the University of Cape Town developed GIFT (Genital InFlammation Test): a relatively inexpensive, cytokine POC rapid test that detects inflammatory cytokine biomarkers of an infection or BV in the female genital tract (36). STIs and BV cause genital inflammation, regardless of other symptoms such as vaginal discharge or genital ulcers being present (6). The measurement of key inflammatory cytokine biomarkers with a rapid POC test can thus potentially identify asymptomatic cases that are otherwise missed and consequently, women who are at an increased risk of acquiring and transmitting HIV (18). The predictive value of the biomarkers has already between validated in multiple cohorts in Africa (26) and the device is to be rolled out in a cross-sectional validation study in Cape Town clinics to evaluate and optimize its performance. It is critical that careful consideration is given to the strategy for GIFT implementation in parallel with syndromic management for women, in consultation with key experts and national stakeholders. Several alternative models for GIFT implementation are being considered: (1) as a test and treat tool to identify asymptomatic women who can then be treated immediately using guidelines similar to those used in the vaginal discharge algorithm; or (2) as a triage tool to identify those needing further etiologic testing (as part of a two-step algorithm) which would reduce the number of etiologic tests that need to be performed. For the purposes of this paper, we considered both of these models.

Although there is a strong body of evidence on the costs and cost-effectiveness of POC testing for STIs, the evidence is more limited in low-resource settings, including Sub-Saharan Africa, especially when considering curable STIs beyond screening for syphilis in antenatal care (37). Furthermore, the cost-effectiveness of this novel test has not yet been established. However, a cost estimation for this intervention at clinics in Cape Town for women aged 15–49 years, as well as a budget impact analysis of implementing it in all primary health facilities across South Africa, has been conducted by Kairu et al. (38). The findings suggested that it could be affordable in the South African context but might not be prioritized given the array of existing and novel interventions and strained health budget. The incremental cost per woman screened for genital inflammation during a family planning visit was estimated to be USD 3.19 at a government-funded clinic, USD 4.16 at a semi-private facility, and USD 4.79 at an NGO-funded facility, not including treatment (costs in 2016 USD). The additional annual expenditure estimates were USD 22,212,636, USD 8,327,176 and USD 7,245,775 in each sector, respectively (costs in 2016 USD). This would amount to up to 17% of the HIV/AIDS budget or 0.85% of the total health budget, when provided at a government health facility. The annual expenditure was based on an estimated coverage rate of 57%, proxied by the contraceptive prevalence rate which reflects the number of women attending family planning clinics. It was then adjusted for the portion of the public attending either the public or private sector. The public sector government clinic covers a much larger portion, approximately 80%, henceforth the much higher estimate (38).

Consequently, a decision analysis model was developed using cost and probability estimates from existing literature and market prices to estimate the cost-effectiveness of five screening and diagnostic strategies for the three highly prevalent curable STIs in South Africa, *C. trachomatis, N. gonorrhoeae* and *T. vaginalis infections*, and BV. These infections were considered for the analysis due to both their high prevalence in South Africa, but mainly their clear association with the increased risk of HIV acquisition and transmission (36). The Health Economic Evaluation Reporting Standards 2022 (CHEERS 2022) has been used to ensure standardized analysis and transparent reporting (39).

## Methods

### Decision analysis model

A static decision tree model (see Appendix A.2) was constructed taking a provider's perspective (South African Department of Health) in Microsoft Excel Office 365 to estimate the cost-effectiveness of five different screening and diagnostic strategies for STIs and BV in women of reproductive age (15–49 years) entering the South African public health sector at primary care level. Economic costs and clinical effectiveness were estimated with the following screening and diagnostic strategies for *C. trachomatis, N. gonorrhoeae, T. vaginalis* and BV:

1. Syndromic management of symptomatic women seeking healthcare (standard of care).
2. Screening all women entering primary care facilities with GIFT followed by management for GIFT-positive cases using a similar antibiotic treatment approach to syndromic management of vaginal discharge syndrome (GIFT-SM).
3. Screening with GIFT (triage approach), followed by testing GIFT-positive cases with GeneXpert NG/CT and GeneXpert TV assays and microscopy (GIFT-Xpert-Microscopy).
4. Screening with GeneXpert (NG/CT and TV) and microscopy alone (Xpert-Microscopy) in health facilities.
5. Screening with gold standard laboratory testing (PCR-Microscopy) at an off-site laboratory.

**Figure 1:**
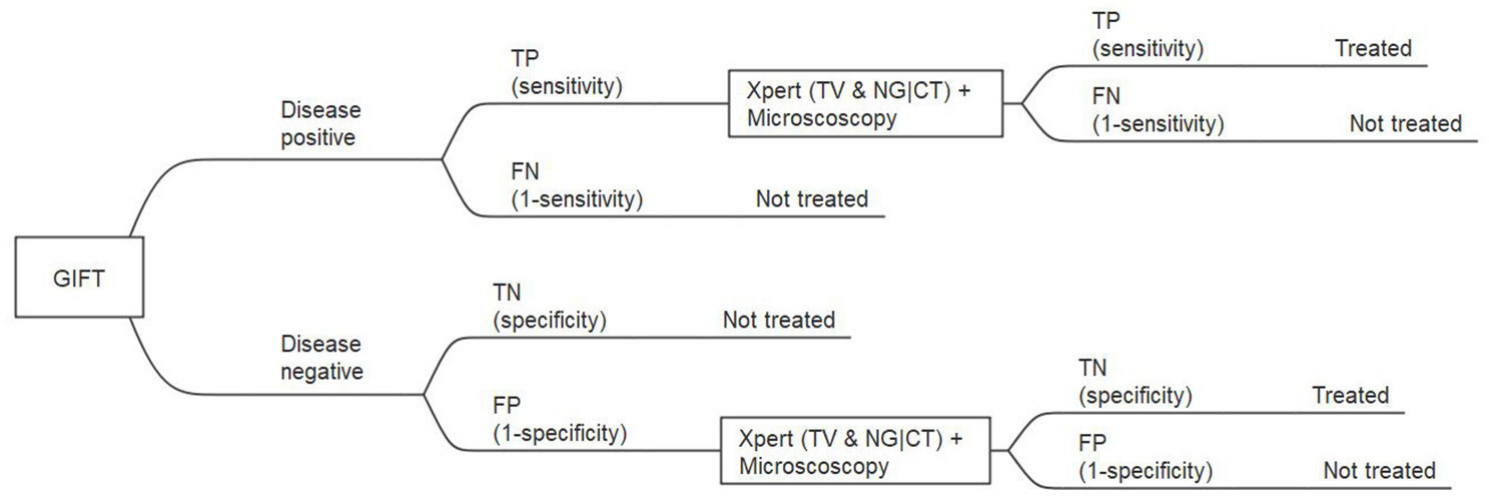
Extract from the full decision tree: GIFT, followed by GeneXpert and microscopy for GIFT positive, subtree. TP, true positive; FP, false positive; TN, true negative; FN, false negative.

The model was constructed for a one-year period only and no discount factor was thus applied to outcomes or costs. The model was populated with the probabilities of events based on estimates from published literature. These strategies were included based on the primary GIFT study protocol and expert opinion on the current circumstances and available technologies in the public health sector (40). Model outcomes were compared using incremental cost-effectiveness ratios (ICERs). In the model, the latter was measured as the additional cost per women diagnosed with an STI(s) and/or BV and put on treatment. Figure 1 shows the sub-tree of the decision tree for model strategy 3: GIFT followed by testing with GeneXpert.

### Data

#### Effectiveness

The effectiveness of each approach was estimated based on the sensitivity and specificity of each approach, with the primary outcome being appropriate diagnosis and treatment administered. The latter was proxied by the sensitivity measure of each diagnostic test or approach. These parameters were obtained from published literature and are shown in Table 1.

**Table 1:** Sensitivity and specificity of diagnostic tests and approaches and model strategies in detecting STIs and BV in reproductive aged women in South Africa.

**Diagnostic test / Approach**	**Sensitivity**	**Specificity**	Missed cases^*^(1-sensitivity)	**Overtreatment (1-specificity)**	**Reference**
**Syndromic management**					
Any discharge causing STI^**^	61.6%	45.4%	38.4%	54.6%	(23)
CT	58.3%	44.7%	41.7%	55.3%	(23)
NG	66.0%	45.2%	34.0%	54.8%	(23)
TV	60.4%	45.6%	39.6%	54.4%	(23)
BV	61.6%	46.0%	38.4%	54.0%	(23)
GIFT for detecting genital inflammation	77.0%	71.0%	23.0%	29.0%	(26)
GeneXpert for detecting CT	100.0%	97.6%	–	2.4%	(41)
GeneXpert for detecting NG	100.0%	100.0%	–	–	(41)
GeneXpert for detecting TV	96.4%	99.6%	3.6%	0.4%	(42)
GeneXpert (average)	99.4%	99.5%	0.6%	0.5%	
Laboratory PCR (CT, NG & TV)	100.0%	100.0%	–	–	(28)
Microscopy (BV)^†^	100.0%	100.0%	–	–	(28)

STI, Sexually transmitted infections; CT, *C. trachomatis*; NG, *N. gonorrhoeae*; TV, *T. vaginalis*.

* Includes asymptomatic cases.

** Includes BV.

† Laboratory- and clinic-based, no difference between the two^*a*^.

a Personal communication: Nigel Garret, 29 May 2019.

Where two or more tests are performed simultaneously in a branch of the decision tree, such as GeneXpert and microscopy, average effectiveness measures were used.

#### Model probabilities

The probability of events in the model was obtained from published literature and are shown in Table 2. The proportion of patients lost to follow-up (LTFU) in the laboratory testing arm was based on published literature related to HIV testing in Africa as no literature on LTFU rates for STI testing exists in these settings (30). The prevalence of BV and the three STIs was based on estimates from South African studies conducted on women in the reproductive age. The overall, average disease prevalence of the three STIs and BV was assumed in the model for simplification.

**Table 2:** Base, low and high estimates of the probability of events in the model.

**Variable**	**Low**	**Base case**	**High**	**Reference**
**Disease prevalence** ^*^				
*C. trachomatis*	4.2%	15.4%	32.8%	(2, 8, 14–17, 43–48)
*N. gonorrhoeae*	1.8%	5.9%	10.9%	
*T. vaginalis*	3.0%	10.8%	20.3%	
Bacterial Vaginosis	33.7%	44.5%	53.0%	
Lost to follow up rate	11.0%	20.0%	49%	(30)

* Based on the average prevalence from 11 studies done in South Africa from 2011 to 2018.

#### Costs

The cost of each subtree of the decision tree was based on estimates obtained from published literatue and market prices from external institutions such as the National Health Laboratory Service (NHLS) and Cepheid. The model incorporated all clinic-level capital and recurrent costs associated with each strategy for diagnosing and treating a single episode of disease. All screening strategies included the cost of a standard clinic visit and treatment costs. In accordance with Kairu et al. (38), no additional programme start-up costs were included for GIFT strategies, expect training of staff to administer the test. Where applicable, it also included the cost of taking a specimen, operating a test assay and obtaining results. Costing was done from the provider's perspective (South African Department of Health) and all costs were inflated to 2019 ZAR and converted to USD based on the average exchange rate for 2019 (USD 1 = R14.32). Unit cost data are presented in Table 3.

**Table 3:** Cost of screening or diagnosis and treatment per patient in 2019 USD.

**Variable**	Cost (USD)^*^	**Reference**
**Syndromic Management**		
Standard clinic visit^**^	13.84	(38)
**Total unit cost**	**13.84**	
**GIFT POC**		
Standard clinic visit^**^	13.84	(38)
Screening with GIFT^***^	3.48	(38)
**Total unit cost**	**17.32**	
**Multiplex PCR and Microscopy (off-site laboratory)**		
Standard clinic visit^¥^	13.84	(38)
Staff time and medical consumables^ψ^	2.55	(38)
Testing with quadruplex PCR^Φ^	30.55	(49)
Testing with laboratory-based microscopy^Φ^	2.98	(49)
Specimen transport^¶^	1.32	(50)
**Total unit cost**	**51.48**	
**GeneXpert and Microscopy (on-site)**		
Standard clinic visit^**^	13.84	(38)
Microscopic examination and testing with GeneXpert^‡^	67.06	[(40), Gwen Stephens (see text footnote ^1^)]
**Total unit cost**	**80.90**	
**Treatment costs**		
Azithromycin (*C. trachomatis)*	0.61	(51)
Ceftriaxone and Azithromycin (*N. gonorrhoeae)*	0.95	(51)
Metronidazole (*T. vaginalis* and BV)	0.11	(51)
Syndrome A treatment^†^	0.91	(51)
Syndrome B treatment^‡^	1.02	(51)

* USD 1= ZAR14.32 (Average exchange rate 2019).

** Includes all clinic level costs of standard clinic visit.

*** Includes cost of test assay, consumables, equipment, training and additional staff time.

ψ Assumed to be same as for GIFT.

Φ Outsourced service, quoted price.

¥ Includes only capital and overheads costs of standard clinic visit, rest included in cost estimate from secondary source.

‡ Includes cost of test assays, consumables, equipment, training and staff time.

¶ For a single specimen transported from the clinic to the NHLS.

† According to South African Department of Health SM Vaginal Discharge algorithm: treat for BV and/or vaginal candidiasis (Appendix A).

‡ According to South African Department of Health SM Vaginal Discharge algorithm: treat for *C. trachomatis, N. gonorrhoeae, T. vaginalis* and Mycoplasma genitalium (Appendix A).

The cost of a family planning visit and examination, as well as screening with GIFT, was based on estimates from Kairu et al. (38). The incremental cost associated with screening with GeneXpert NG/CT and TV assays was mainly based on study by Stime et al. (40). The cost of a standard family planning clinic visit was added to this estimate since this was not included by Stime et al. (40). The cost of laboratory testing was mainly obtained from the quoted NHLS price since service is outsourced from the Department of Health. The total unit cost, however, includes the cost of a standard family planning clinic visit, collection of specimens as well as the transport cost of two specimens.

The treatment regimens for syndromic management, for strategies 1 and 2, were based on the guidelines for STI treatment in South Africa. Given that syndromic management is mainly followed in the public health sector, guidelines do not specify treatment regimens for individual infections, but rather for groups of infections with similar symptoms (see guidelines in Appendix A.1). Therefore, drug regimens for individual infections, for strategies 3–5, were obtained from (41). Drug costs were obtained from the latest available master procurement catalog of the Western Cape Department of Health at the time of analysis (April 2019). In the based case scenario, the average treatment cost was assumed for simplification.

## Model assumptions

Only women in their reproductive age, ages 15–49 years, were included following what was done for the GIFT costing study (38). This age group typically carries the highest burden of social and psychological consequences of STIs and are typically most susceptible to STIs and BV (52). For syndromic management, it was assumed that all women who have symptoms seek care and accept consequent treatment. All screening strategies were based on opportunistic screening of any woman entering a primary healthcare facility. It was assumed that all women accept the screening tests and resulting treatment when administered.

In addition, several assumptions were made when using and adapting relevant data from secondary sources. Firstly, the effectiveness of GIFT was based on the sensitivity and specificity found in the biomarker validation study, as it been established in the field. The prevalence of diseases for the study population was based on the average estimates from 11 studies conducted in South Africa from 2011 to 2018. These studies were all based in different study settings and included different age groups, although within the bounds of 15–49 years, and they followed varying research methodologies. The probabilities of the four diseases were then averaged to arrive at a single estimate. It was also assumed that all women who are symptomatic seek care and all who are offered a screening test, would accept it.

Cost parameters were also collected from varying sources. A standard clinic visit was included for all model comparators, for which the costs were extracted from (38). Across non-GIFT strategies, this was then combined with cost estimates from other relevant sources. Cost sources for the different model comparators also differed in the extent to which parameters were compounded and methods used for estimation. The costing of staff time and the inclusion of consumables were thus not standard across cost estimates. Furthermore, laboratory testing was cost based on a single quoted price received from the NHLS, while for GeneXpert, Microscopy and GIFT unit costs were available.

### Sensitivity analysis

We performed one-way sensitivity analyses to establish the robustness of the results given the high variability of estimates found in literature, uncertainties in the data and the assumptions made in the model. Table 4 contains results from the base case cost-effectiveness scenario. Key parameters were varied across a reasonable range while holding all other parameters constant at their base case values. These parameters included GIFT test cost, staff time, GeneXpert test costs, GIFT sensitivity, lost to follow-up rates, disease prevalence, health-seeking behavior and test acceptance rates and syndromic management sensitivity. Parameter values were varied based on relevant values from the literature. Refer to Table 5 for details on how variables were varied in the sensitivity analysis.

**Table 4:** Base case cost-effectiveness results in 2019 USD.

**Approach**	Cost (USD)^*^	**Incremental cost (USD)**	Effectiveness^**^	**Incremental effectiveness**	**ICER (USD)^‡^**	**Missed cases (1-sensitivity)**	**Overtreatment (1-specificity)**
Syndromic Management	14.23		0.46		–	54.40%	38.43%
GIFT-SM	16.66	2.43	0.77	0.31	11.08	23,00%	29,00%
GIFT-Xpert-Microscopy	40.05	25.83	0.77^#^	0.31^¶^	92.99^¥^	23.46%	0.14%
PCR-Microscopy	51.54	37.32	0.80	0.34	108.48	20.00%	–
Xpert-Microscopy	80.89	66.76	0.99	0.53	125.49	1,20%	0.47%

* Cost per women to diagnose and treat a single episode of disease.

** Based on combined sensitivity of each approach.

# 0.765.

¶ 0.309.

‡ Incremental cost divided by incremental effectiveness; Incremental to standard of care (Syndromic Management).

¥ Eliminated through absolute dominance.

**Table 5:** The impact of one-way sensitivity analyses on the cost-effectiveness results in 2019 USD.

	**Incremental cost-effectiveness (USD)**			
	**GIFT-SM**	**GIFT-Xpert-Microscopy**	**PCR-Microscopy**	**Xpert-Microscopy**
Base case	11.08	Dominated^*^	108.48	125.49
**Cost parameters**				
**GIFT test assay**				
+50%	11.60	Dominated^*^	108.48	125.49
−50%	10.56	Dominated^*^	108.48	125.49
**GIFT staff time**				
+50%	13.46	Dominated^*^	108.48	125.49
+30%	12.51	Dominated^*^	108.48	125.49
**GeneXpert test assays**				
+50%	11.08	Dominated^*^	108.48	164.54
−50%	11.08	Dominated^*^	Dominated^*^	86.43
**Probabilities**				
**GIFT sensitivity**				
100%	6.48	Dominated^*^	Dominated^*^	Dominated^*^
50%	77.87	Dominated^*^	108.48	125.49
**Disease probabilities**				
11%	10.97	Dominated^*^	108.41	125.43
29%	11.21	Dominated^*^	108.56	125.56
**LTFU**				
11%	11.08	Dominated^*^	86.00	125.49
49%	11.08	Dominated^*^	Dominated^*^	125.49
**Syndromic management sensitivity**				
+50%	39.96	Dominated^*^	Dominated^*^	219.46
−50%	6.50	Dominated^*^	65.31	87.90
**Patient behavior**				
**Symptomatic women seeking care**				
58.8%^**^	14.67	Dominated^*^	111.76	127.61
70%	13.69	Dominated^*^	110.87	127.03
**Acceptance of screening test**				
18.9%	2.09	Dominated^*^	108.48	125.49
25%	2.77	Dominated^*^	108.48	125.49
50%	5.54	Dominated^*^	108.48	125.49
70%	7.75	Dominated^*^	108.48	125.49

* Eliminated through absolute dominance.

** Survey included both male and female school learners.

We also performed a threshold analysis for GIFT sensitivity. Lastly, four alternative decision trees in which the decision analysis was modeled for each disease separately was constructed to test the average disease probability and treatment costs assumptions made in the base case decision tree.

## Results

### Base-case scenario

Screening with GIFT-SM was the most cost-effective strategy in the base case scenario with an ICER of USD 11.08 per women diagnosed and put on treatment. GIFT-Xpert-Microscopy, with an ICER of USD 92.99, was absolutely dominated (more costly but less effective than the previous less costly alternative) and thus eliminated from the decision problem. Extended dominance, on the other hand, is when an alternative is ruled out because it is equally effective, but more costly or vice versa (53). The remaining two strategies were also potentially cost-effective, but with much higher ICERs than GIFT-SM of USD 108.48 and USD 125.49, respectively.

**Figure 2:**
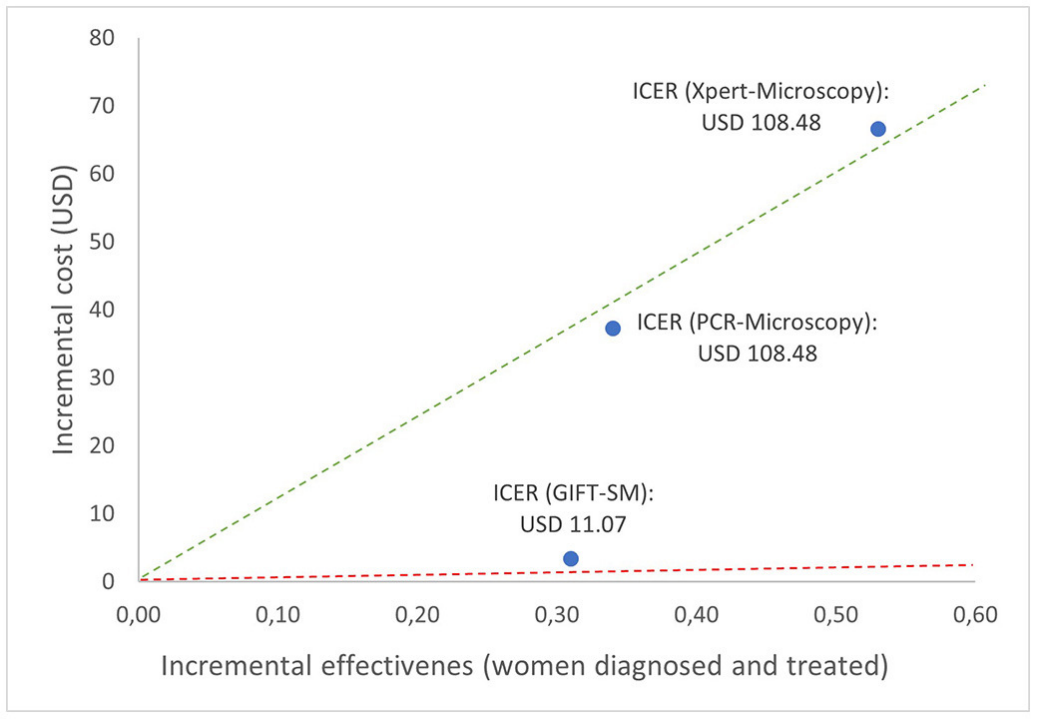
Cost-effectiveness plane depicting base case ICERs and hypothetical cost-effectiveness thresholds.

The relationship between the incremental cost and effectiveness of each alternative and three hypothetical thresholds is shown in Figure 2. All the ICERs appear in the first quadrant, revealing that each comparator presents a possibly cost-effective option; being both more effective but also more costly than syndromic management; the comparator. The figure graphically shows that GIFT-SM is the most cost-effective of the three, having the lowest ICER. To make inference about the absolute cost-effectiveness of alternatives in the decision-making context, a cost-effectiveness- or willingness-to-pay (WTP) threshold is required. For a threshold lower than USD 11.08 (red dotted line), none of the options would be cost-effective. Similarly, for a threshold above USD 108.48 but below USD 125.49 (green dotted line) both GIFT-SM and PCR-Microscopy would be cost-effective.

Screening with GIFT would give an indication of genital inflammation only, but not the exact infection causing inflammation. As such, it would not be possible to tailor treatment directly to an etiologic agent based on a GIFT positive test so specificity of treatment would not be improved over syndromic management, and there is a risk for overtreatment as there is for syndromic management. However, the overall specificity of GIFT for identifying women with any STI and/or BV is higher than syndromic management, thus the proportion of women receiving treatment even though they do not have an STI or BV would be lower for GIFT. The GIFT strategies, however, show improvement in terms of identifying cases that are typically missed under the current standard of care (23% compared to 89.5%), albeit not as effective as PCR, Xpert and microscopy. A triage algorithm for GIFT, linking GIFT positive cases to etiological testing would overcome some of these challenges but increase cost.

### Sensitivity analysis

The cost of the GIFT test assay was varied 50% both ways to account for possible fluctuations in the current quoted prices since manufacturing is still underway. This rendered similar ICERs of GIFT-SM to the base-case scenario with no changes in the relative cost-effectiveness observed.

To account for possible underestimation of staff time costs, given that GIFT has not been validated in a real-world setting, this cost related to the GIFT strategies was varied upward across a reasonable range. These variations revealed that GIFT-SM becomes less cost-effective as staff costs are increased but that it remained the most cost-effective strategy in the model.

The cost of the GeneXpert test assays and machine was also varied simultaneously, both 50% upward and downward to account for exchange rate variability (since the cost is based on prices quoted in USD) and the possibility that the GeneXpert machines can in some cases be provided to state institutions at subsidized prices.^1^ With the upward variation, the relative cost-effectiveness of the model strategies remained unchanged while Xpert-Microscopy dominated PCR-Microscopy when the parameters were decreased with 50%. In the latter case, GIFT-SM remained the most cost-effective strategy, however.

Since the field-ready GIFT device has not yet been validated in a clinical study, the base-case sensitivity of the device is estimated from findings from the biomarker validation study (54). Therefore, it is possible that the device might have a different sensitivity in practice. GIFT sensitivity was consequently decreased to 50% and increased to 100% to test this limitation. At 50% sensitivity the relative cost-effectiveness of the comparators remained unchanged, although the ICER of GIFT-SM increased substantially to USD 77.8. At 100% sensitivity, GIFT-SM was only cost-effective model comparator. Evidently, this parameter affects the cost-effectiveness conclusions and the absolute cost-effectiveness of GIFT significantly and will thus affect whether it would be considered a good investment, or priority, in comparison to existing or other novel interventions.

STI/BV prevalence and LTFU were varied according to the highest and lowest estimates found in the literature. At both 11 and 29% average STI/BV prevalence, the ICERs of each model comparator did not change significantly from the base-case scenario and the cost-effectiveness conclusions remained the same. When the LTFU was varied to 49%, both GIFT-Xpert-Microscopy and PCR-Microscopy were eliminated through absolute dominance, but GIFT-SM remained the most cost-effective. At the lowest LTFU estimate, 11%, the relative cost-effectiveness of all the model strategies remained unchanged.

To account for uncertainty with regards to the effectiveness of syndromic management in the South African setting, the sensitivity of the approach was varied with both 50% upward, and downward. With the downward adjustment, the GIFT-Xpert-Microscopy was still dominated, and the relative cost-effectiveness of the comparators remained unchanged although each ICER lowered substantially with the ICER of GIFT-SM lowering to USD 6.50. When adjusted upward, PCR-Microscopy was also dominated and the remaining two ICERs, of GIFT-SM and Xpert-Microscopy, increased substantially with GIFT-SM presenting an ICER of USD 39.96.

Our model assumed that all women who have symptoms seek care under syndromic management and that all women who are screened accept the test administered to them. These assumptions were tested based on other studies and estimates found in the literature. Health care-seeking behavior among women in South Africa remains limited and is typically more compromised in rural compared to urban settings (55). Estimates from two sources were used to test the health care seeking assumptions with further one-way sensitivity analyses. Firstly, Sahin-Hodoglugil et al. (55) produced a baseline estimate of 70% of women seeking care for STIs. Secondly, the National Survey on youth risk behavior was utilized. This survey revealed that 58.8% of the study population received treatment for a known STI (56). These variations did not change the relative cost-effectiveness of any of the strategies and GIFT-Xpert-Microscopy remained dominated. It did, however, produce slightly higher ICERs for the remaining strategies than in the base-case analysis, due to a decrease in the cost of the baseline strategy; syndromic management.

The assumption on screening acceptability was tested by introducing this parameter to the model and varying it based on estimates used in two studies conducted in the United Kingdom, due to the lack of reliable local estimates available for this study setting (57, 58). Lower acceptability resulted in lower GIFT-SM ICERs. This analysis, however, results in a paradox since lower acceptance rates increases the cost-effectiveness of all three strategies, but in reality, fewer health outcomes would be realized if fewer women agree to be screened, as in these scenarios. This is due to the structure of the model. Care should be taken in judging these estimates. To optimize any screening intervention, acceptance rates in the setting should be established and considered in its design.

A single threshold analysis revealed that the GIFT+SM strategy would remain the most cost-effective unless the sensitivity of GIFT were to decrease to below 48.76%. The individual decision tree analyses (Table 6) revealed that GIFT-SM consistently remained the most cost-effective strategies when looking at the diseases individually. See Appendix A.3 for individual model parameters.

**Table 6:** Results from separated decision trees in 2019 USD.

**BV**	**Incremental Cost (USD)**	**Incremental effectiveness**	**ICER (USD)**
Syndromic management	–	–	–
GIFT-SM	3.94	0.31	12.71
GIFT-Xpert-Microscopy	37.26	0.31	^*^
PCR-Microscopy	37.63	0.54	69.68
Xpert-Microscopy	67.06	0.54	^*^
**CT**	**Incremental Cost (USD)**	**Incremental effectiveness**	**ICER (USD)**
Syndromic management	–	–	–
GIFT-SM	3.59	0.63	5.69
GIFT-Xpert-Microscopy	27.69	0.63	^*^
PCR-Microscopy	37.42	0.66	56.61
Xpert-Microscopy	66.91	0.86	77.71
**NG**	**Incremental Cost (USD)**	**Incremental effectiveness**	**ICER (USD)**
Syndromic management	–	–	–
GIFT-SM	1.04	0.59	1.76
GIFT-Xpert-Microscopy	22.10	0.59	^*^
PCR-Microscopy	35.26	0.62	56.78
Xpert-Microscopy	54.95	0.82	66.94
**TV**	**Incremental Cost (USD)**	**Incremental effectiveness**	**ICER (USD)**
Syndromic management	–	–	–
GIFT-SM	3.78	0.59	6.39
GIFT-Xpert-Microscopy	26.37	0.56	^**^
PCR-Microscopy	37.60	0.62	60.55
Xpert-Microscopy	67.03	0.79	85.38

* Eliminated through extended dominance.

** Eliminated through absolute dominance.

## Discussion

Under the current standard of care, a significant proportion of women with STIs and/or BV remain untreated, particularly when using the vaginal discharge algorithms of syndromic management largely due to the frequently asymptomatic nature of these infections. In our model, we compared the cost and effectiveness of four alternative STI and BV management strategies to this standard of care. These strategies were modeled for women aged 15–49 years.

The results from the economic evaluation suggest that the introduction of at least an annual screening for genital inflammation with the rapid GIFT POC test for the target population into the South African public health sector would be the most cost-effective way to improve case detection of STIs and BV, relative to other model comparators. Given the interaction with HIV risk, reducing this burden could also significantly reduce HIV transmission and new infections. The introduction of this screening approach could also identify high-risk women who can be linked to pre-exposure prophylaxis programs for the prevention of HIV. Early treatment and prevention of curable STIs such as chlamydia and gonorrhea can lead to significant health gains in a population (55).

There is no standard cost-effectiveness threshold in South Africa but there have been attempts to estimate this. Woods et al. estimated a threshold of USD 1,175–4,714 (in 2013 prices) per disability-adjusted life year (DALY) averted (59), while Meyer-Rath et al. estimated a WTP threshold of USD 547–872 per life year saved (60). Edoka and Stacey estimated a threshold of ~USD 3,015 per DALY averted (in 2015 prices) (61). However, the results of this study cannot be directly compared to these thresholds due to the lack of a mutual ICER comparator. No definite conclusion on the absolute cost-effectiveness can thus be made. Nonetheless, an ICER of USD 11.08 per additional woman diagnosed and treated appears low in the South African context.

The robustness of the cost-effectiveness results was tested in various sensitivity analyses. The conclusion remained the same in all the analyses with the ICER of GIFT-SM only increasing or decreasing marginally and additionally, PCR-Microscopy eliminated by dominance in four cases and Xpert-Microscopy in one case. From the sensitivity analysis, testing the acceptance of GIFT screening by women arises as an opportunity for further study during the GIFT pilot study or other research in order to truly establish whether the approach could improve STI management or not. Furthermore, establishing the effectiveness of GIFT in the field will be key in judging the appropriateness of the intervention.

Given that the field performance of GIFT-SM in terms of overtreatment (if the test and treat model is considered) is yet to be determined, it may be that PCR-Microscopy should be considered in high-risk or high-burdened settings to enhance STI management rather than test-and-treat. However, although GIFT-SM is the least desirable in terms of overtreatment apart from syndromic management (see Table 1), Passmore et al. and Lennard et al. note that the other infections present in women that typically lead to a false positive result with GIFT, such as *Atopobuim, Prevotella, Shuttleworthia* and *Aerococcus*, are not detected by gold standard STI and BV tests (62, 63). These organisms would, however, also respond to the antibiotics administered. This would result in unintended health gains not measured in this analysis.

From a budget impact perspective, Kairu et al. (38) found that the national roll-out of an annual, opportunistic screening intervention could be feasible, but that it would have to be considered carefully given the trade-offs that would have to be made within the health budget to avail the required funds.

To limit start-up costs, GIFT screening could be introduced alongside regular family planning visits, antenatal visits or HIV testing programmes—key initiatives set out in South Africa's National Strategic Plan for HIV, TB and STIs 2017–2020 (32). Alternatively, key populations could be identified for whom the approach should be initiated. Such populations could include women at high risk for STIs such as sex workers, pregnant women, HIV-positive persons, younger women or women residing in settings with known high disease prevalence. In this case, register-based screening (target individuals are identified through existing databases) could be useful or key screening questions could be set up to identify these individuals as they enter healthcare (opportunistic screening). Information system challenges might arise, however, and additional costs might arise where systems would need to be created or improved to ensure adequate record-keeping of screening history or to identify high-risk individuals.

The study faced various challenges and limitations, with the simplicity of the model of choice due to data constraints and the use of secondary data from varying sources likely being the most significant. As part of the first clinical study on the field-ready GIFT device, this early cost-effectiveness model will be expanded and more complex modeling will be explored, including incorporating DALYs as outcome measure. The model was designed as a static, decision tree analysis that did not include any sequelae or long-term complications typically associated with STIs and BV nor did we factor in persistence, recurrence or re-infections. This might likely have led to the cost of having an STI or BV being underestimated. Adverse drug events were also not included due to the short-term nature of the analysis and would likely have underestimated the cost of treatment. On the other hand, the model did not include the potential benefits of HIV prevention through STI and BV treatment or other positive effects of increased diagnosis and treatment on STI/BV prevalence in the population over time. This may have led to underestimation of effectiveness of the screening interventions. To establish a more sophisticated, dynamic model that would be able to inform decision-making more comprehensively, future research should focus on estimating these parameters.

Furthermore, the effectiveness of GIFT was assumed to be the sensitivity and specificity found in a biomarker validation study, which may not be a true representation of the effectiveness that would be yielded when implemented in the field. Although this assumption was tested in the sensitivity analysis, implementation challenges that may arise in the field may influence the model outcomes more than captured in the analysis.

## Conclusions

Although syndromic management remains the most affordable approach to STI/BV care, it is not adequately dealing with the massive STI and BV disease burden faced by South African women. Screening with gold standard or more rapid tests such as GeneXpert and microscopy, on the other hand, provide more desirable health outcomes in terms of women diagnosed and treated and limit overtreatment, but these diagnostics remain expensive, while GeneXpert is considered “near” POC rather than POC because of the instrumentation needed. The results from the economic evaluation suggest that the introduction of screening with the rapid GIFT device in the South African public health sector would be the most cost-effective way to improve STIs and BV care in women while simultaneously having positive effects on the HIV-epidemic. This analysis presents a first step in establishing the cost-effectiveness of the various screening approaches. It reveals that further research should be done to evaluate the feasibility of different implementation options within this resource-constrained setting. To enable the establishment of a more comprehensive and dynamic model, future studies should focus on quantifying the effect that screening for STIs and BV would have on averted HIV cases and STI disease probability. Furthermore, it would be important to consider its consequent impact on the outcomes of the decision model to enable a more comprehensive decision beyond simply affordability.

## Data availability statement

The original contributions presented in the study are included in the article/Supplementary material, further inquiries can be directed to the corresponding author.

## Ethics statement

The study was approved by University of Cape Town Human Research Ethics Committee (HREC).

## Author contributions

ESm analyzed and interpreted the cost and effectiveness data for the model comparators and was the major contributor in writing the manuscript. ESi contributed in study design, data analysis, and interpretation of the findings. LM contributed in study design, interpretation of the findings, and manuscript preparation. J-AP contributed to interpretation of findings in the final manuscript. All authors read and approved the final manuscript.

## Footnotes

^1^Personal communication: Gwen Stephens (Cepheid), 3 June 2019.

## References

[1] World Health Organization. SEXUALLY TRANSMITTED INFECTIONS (STIs): The Importance of a Renewed Commitment to STI Prevention and Control in Achieving Global Sexual and Reproductive Health. (2012). Geneva, Switzerland: World Health Organization.

[2] van der Eem L Dubbink JH Struthers HE McIntyre JA Ouburg S Morré SA. Evaluation of syndromic management guidelines for treatment of sexually transmitted infections in South African women. Trop Med Int Health. (2016) 21:1138–46. 10.1111/tmi.12742 27350659

[3] Cohen CR Lingappa JR Baeten JM Ngayo MO Spiegel CA Hong T. et al. Bacterial vaginosis associated with increased risk of female-to-male HIV-1 transmission : a prospective cohort analysis among African couples. PLoS Med. (2012) 9:1–9. 10.1371/journal.pmed.1001251 22745608 PMC3383741

[4] Kamb M Lackritz E Mark J Jackson D Andrews H. Sexually Transmitted Infections in Developing Countries: Current Concepts and Strategies on Improving STI Prevention Treatment and Control. (2008). Geneva, Switzerland: World Health Organization.

[5] Chesson HW Mayaud P Aral SO. Sexually Transmitted Infections: Impact and Cost-Effectiveness of Prevention. In:Holmes K Bertozzi S Bloom B Jha P, editors. Disease Control Priorities: Major Infectious Diseases. Third edit. Washington, DC: World Bank (2017). p. 2003–232. 30212101

[6] Masson L Arnold KB Little F Mlisana K Lewis DA Mkhize N. Inflammatory cytokine biomarkers to identify women with asymptomatic sexually transmitted infections and bacterial vaginosis who are at high risk of HIV infection. Sex Transm Infect. (2016) 92:186–93. 10.1136/sextrans-2015-052072 26511781 PMC6801014

[7] Mlisana KP. The impact of sexually transmitted infections (STI) and genital tract inflammation on HIV-1 acquisition and rate of disease progression in subtype C infected women. [Doctor of Philosophy (Medicine)]. University of KwaZulu-Natal (2014).

[8] Johnson LF Dorrington RE Bradshaw D Coetzee DJ. The effect of syndromic management interventions on the prevalence of sexually transmitted infections in South Africa. Sex Reprod Healthcare. (2011) 2:13–20. 10.1016/j.srhc.2010.08.006 21147454

[9] United Nations,. United Nations Programme on HIV/AIDS report on the on the global AIDS epidemic. (2010). Available from: http://www.unaids.org (accessed July 9, 2018).

[10] Johnson L Bradshaw D Dorrington R Comparative A Assessment R Group C. The burden of disease attributable to sexually transmitted infections in South Africa in 2000. S Afr Med J. (2007) 97:658–62. Available online at: https://www.ajol.info/index.php/samj/article/view/13895 17952222

[11] Mahlati P, Dlamini, J,. Minimum Data Sets for Human Resources the Surgical Workforce in South Africa: A rapid analysis of stock migration. African Institute for Health Leadership Development. (2015). p. 3–5. Available from:https://pdf4pro.com/amp/download?data_id=768529&slug=minimum-data-sets-for-human-resources-for

[12] World Health Organization. Guidelines for the management of sexually transmitted infections. (2003). Available from: https://apps.who.int/iris/bitstream/handle/10665/42782/9241546263_eng.pdf;jsessionid=444F248FE25D6F14C09AA93FADB78707?sequence=1 34370424

[13] Kettler H White K Hawkes S. Mapping the Landscape of Diagnostics for Sexually Transmitted Infections: Key Findings and Recommendations. (2004). Geneva, Switzerland: World Health Organization.

[14] Kaida A Dietrich JJ Laher F Beksinska M Jaggernath M Bardsley M. A high burden of asymptomatic genital tract infections undermines the syndromic management approach among adolescents and young adults in South Africa: Implications for HIV prevention efforts. BMC Infect Dis. (2018) 18:1–11. 10.1186/s12879-018-3380-6 30285705 PMC6171143

[15] Francis S Mthiyane N Baisley K Mchunu L Ferguson J Smit T. Prevalence of sexually transmitted infections among young people in South Africa: A nested survey in a health and demographic surveillance site. PLoS Med. (2018) 790:1–26. 10.1371/journal.pmed.1002512 29485985 PMC5828358

[16] Barnabas SL Dabee S Passmore JAS Jaspan HB Lewis DA Jaumdally SZ. Converging epidemics of sexually transmitted infections and bacterial vaginosis in southern African female adolescents at risk of HIV. Int J STD AIDS. (2018) 29:531–9. 10.1177/0956462417740487 29198180

[17] Mlisana K Naicker N Werner L Roberts L van Loggerenberg F Baxter C. Symptomatic vaginal discharge is a poor predictor of sexually transmitted infections and genital tract inflammation in high-risk women in South Africa. J Infect Dis. (2012) 206:6–14. 10.1093/infdis/jis298 22517910 PMC3490689

[18] Masson L Passmore JAS Liebenberg LJ Werner L Baxter C Arnold KB. Genital Inflammation and the Risk of HIV Acquisition in Women. Clin Infect Dis. (2015) 61:260–9. 10.1093/cid/civ298 25900168 PMC4565995

[19] Wajid A. Syndromic management of sexually transmitted infections: Is it time for the World Health Organization to revise its algorithms? Global J Med Public Health. (2015) 4:3–4.

[20] Ramkissoon A Kleinschmidt I Beksinska M Smit J Hlazo J Mabude Z. National baseline assessment of sexually transmitted infection and HIV services in South African public sector health facilities. Durban: Reproductive Health Research Unit (2004). Available from: http://www.rhru.co.za/

[21] Reagon G Irlam J Levin J. The National Primary Health Care Facilities Survey 2003. Durban: Health Systems Trust (2004).

[22] Unemo M Bradshaw CS Hocking JS de Vries HJC Francis SC Mabey D. Sexually transmitted infections: challenges ahead. Lancet Infect Dis. (2017) 17:e235–79. 10.1016/S1473-3099(17)30310-9 28701272

[23] Verwijs MC Agaba SK Sumanyi JC Umulisa MM Mwambarangwe L Musengamana V. Targeted point-of-care testing compared with syndromic management of urogenital infections in women (WISH): a cross-sectional screening and diagnostic accuracy study. Lancet Infect Dis. (2019) 19:658–69. 10.1016/S1473-3099(18)30724-2 31031172

[24] Mayaud P Mccormick D. Interventions against sexually transmitted infections (STI) to prevent HIV infection. Br Med Bull. (2001) 58:129–53. 10.1093/bmb/58.1.129 11714628

[25] Vickerman P. Modelling the cost effectiveness of rapid point of care diagnostic tests for the control of HIV and other sexually transmitted infections among female sex workers. Sex Transm Infect. (2006) 82:403–12. 10.1136/sti.2006.020107 17012515 PMC2563843

[26] Masson L Barnabas S Deese J Lennard K Dabee S Gamieldien H. Inflammatory cytokine biomarkers of asymptomatic sexually transmitted infections and vaginal dysbiosis: A multicentre validation study. Sex Transm Infect. (2019) 95:5–12. 10.1136/sextrans-2017-053506 30018088

[27] Workowski KA Bolan GA. Sexually Transmitted Diseases Treatment Guidelines, 2015. MMWR Recomm Rep. (2015) 64:1–137. Available online at: https://www.cdc.gov/mmwr/pdf/rr/rr6403.pdf PMC5885289 26042815

[28] Unemo M Ballard R Ison C Lewis D Ndowa F Rosanna P. Laboratory diagnosis of sexually transmitted infections, including human immunodeficiency virus. Geneva, Switzerland: World Health Organization (2013), 244. Available from: http://www.fidssa.co.za/images/LR_WHO_lab_manual_2013.pdf

[29] Brotman RM Klebanoff MA Nansel TR Yu KF Andrews WW Zhang J. Bacterial vaginosis assessed by gram stain and diminished colonization resistance to incident gonococcal, chlamydial, and trichomonal genital infection. J Infect Dis. (2010) 202:1907–15. 10.1086/657320 21067371 PMC3053135

[30] Obermeyer CM Osborn M. The utilization of testing and counseling for HIV: A review of the social and behavioral evidence. Am J Public Health. (2007) 97:1762–74. 10.2105/AJPH.2006.096263 17761565 PMC1994175

[31] World Health Organization. Sexually Transmitted Infections (STIs). World Health Organization (2021). Available online at: https://www.who.int/news-room/fact-sheets/detail/sexually-transmitted-infections-(stis) (accessed September 12, 2021).

[32] SANAC. South Africa's National Strategic Plan for HIV, TB and STIs 2017-2022. (2017). Available from: http://sanac.org.za/2017/05/11/download-the-full-version-of-the-national-strategic-plan-for-hiv-tb-and-stis-2017-2022/ (accessed August 21, 2019).

[33] Western Cape Government Health. Provincial Strategic Plan on HIV/AIDS, STIs and TB. (2016), 1–132. Available from: https://www.westerncape.gov.za/assets/departments/health/burden_of_disease_update_ncds_.pdf (accessed August 21, 2019).

[34] Gaydos C Hardick J. Point of care diagnostics for sexually transmitted infections: perspectives and advances. Expert Rev Anti Infect Ther. (2014) 12:657–72. 10.1586/14787210.2014.880651 24484215 PMC4065592

[35] Gaydos CA van der Pol B Jett-Goheen M Barnes M Quinn N Clark C. Performance of the cepheid CT/NG Xpert rapid PCR test for detection of Chlamydia trachomatis and Neisseria gonorrhoeae. J Clin Microbiol. (2013) 57:1666–72. 10.1128/JCM.03461-12 23467600 PMC3716060

[36] Masson L Mlisana K Little F Werner L Mkhize NN Ronacher K. Defining genital tract cytokine signatures of sexually transmitted infections and bacterial vaginosis in women at high risk of HIV infection: A cross-sectional study. Sex Transm Infect. (2014) 90:580–7. 10.1136/sextrans-2014-051601 25107710

[37] Saweri OPM Batura N Adawiyah R. al, Causer L, Pomat W, Vallely A, et al. Cost and cost-effectiveness of point-of-care testing and treatment for sexually transmitted and genital infections in pregnancy in low-income and middle-income countries: A systematic review protocol. BMJ Open. (2019) 9:1–5. 10.1136/bmjopen-2019-029945 31727649 PMC6887066

[38] Kairu A Masson L Passmore JAS Cunnama L Sinanovic E. Rapid point-of-care testing for genital tract inflammatory cytokine biomarkers to diagnose asymptomatic sexually transmitted infections and bacterial vaginosis in women: cost estimation and budget impact analysis. Sex Transm Dis. (2022) 49:237–43. 10.1097/OLQ.0000000000001565 34596633 PMC8820766

[39] Husereau D Drummond M Augustovski F Chaiyakunapruk N Greenberg D Loder E. Consolidated Health Economic Evaluation Reporting Standards (CHEERS) 2022 Explanation and Elaboration: A Report of the ISPOR CHEERS II Good Practices Task Force. Value Health. (2021) 25:10–31. 10.1016/j.jval.2021.10.008 35031088

[40] Stime KJ Drain PK Mindel A Panday S Ngobese H Ngomane N. The cost of diagnostic versus syndromic management of sexually transmitted infections in the hiv epicentre. Sex Transm Infect. (2017) 93:A211. 10.1136/sextrans-2017-053264.549

[41] Garrett N Mitchev N Osman F Naidoo J Dorward J Singh R. Diagnostic accuracy of the Xpert CT/NG and OSOM Trichomonas Rapid assays for point-of-care STI testing among young women in South Africa: A cross-sectional study. BMJ Open. (2019) 9:1–5. 10.1136/bmjopen-2018-026888 30782948 PMC6367982

[42] Gaydos CA Klausner JD Pai NP Kelly H Coltart C Peeling RW. Vaginalis in Women and Men. Sex Transm Infect. (2017) 93:S31–5. 10.1136/sextrans-2016-053063 28684611 PMC5723541

[43] Torrone EA Morrison CS Chen PL Kwok C Francis SC Hayes RJ. Prevalence of sexually transmitted infections and bacterial vaginosis among women in sub-Saharan Africa: An individual participant data meta-analysis of 18 HIV prevention studies. PLoS Med. (2018) 15:1–38. 10.1371/journal.pmed.1002511 29485986 PMC5828349

[44] Giuliano AR Botha MH Zeier M Abrahamsen ME Glashoff RH van der Laan LE. High HIV, HPV, and STI prevalence among young Western Cape, South African women: EVRI HIV prevention preparedness trial. J Acquir Immune Defic Syndr. 1988. (2015) 68:227–35. 10.1097/QAI.0000000000000425 25415290 PMC4378717

[45] Garrett NJ Osman F Maharaj B Naicker N Gibbs A Norman E. Beyond syndromic management: Opportunities for diagnosis-based treatment of sexually transmitted infections in low- and middle-income countries. PLoS ONE. (2018) 13:e0196209. 10.1371/journal.pone.0196209 29689080 PMC5918163

[46] Kularatne RS Niit R Rowley J Kufa-Chakezha T Peters RPH Taylor MM. Adult gonorrhea, chlamydia and syphilis prevalence, incidence, treatment and syndromic case reporting in South Africa: Estimates using the Spectrum-STI model, 1990-2017. PLoS ||. (2018) 13:1–22. 10.1371/journal.pone.0205863 30321236 PMC6188893

[47] Abbai NS Wand H Ramjee G. Sexually Transmitted Infections in Women Participating in a Biomedical Intervention Trial in Durban: Prevalence, Coinfections, and Risk Factors. J Sex Transm Dis. (2013) 2013:1–6. 10.1155/2013/358402 26316957 PMC4436868

[48] de Waaij DJ Dubbink JH Ouburg S Peters RPH Morré SA. Prevalence of Trichomonas vaginalis infection and protozoan load in South African women: A cross-sectional study. BMJ Open. (2017) 7:1–6. 10.1136/bmjopen-2017-016959 28993385 PMC5640031

[49] National Health Laboratory Service. NHLS State Price List 2018. (2018). p. 1–24. Durban, South Africa: National Health Laboratory Service.

[50] Cunnama L Sinanovic E Ramma L Foster N Berrie L Stevens W. Using Top-down and Bottom-up Costing Approaches in LMICs: the case for using both to assess the incremental costs of new technologies at scale. Health Econ. (2016) 25:53–66. 10.1002/hec.3295 26763594 PMC5066665

[51] Department of Health. Master Procurement Catalogue. (2019). Available from: http://www.health.gov.za/index.php/component/phocadownload/category/196 (accessed on April 1, 2019).

[52] World Health Organization. Global Health Sector Strategy on Sexually Transmitted Infections. 2016–2021: Towards Ending STIs. Geneva, Switzerland*:* World Health Organization (2016).

[53] Drummond MF Sculpher MJ Claxton K Stoddart GL Torrance GW. Methods for the economic evaluation of health care programmes. Oxford, UK: Oxford University Press (2015).

[54] McKinnon LR Liebenberg LJP Yende-Zuma N Archary D Ngcapu S Sivro A. Genital inflammation undermines the effectiveness of tenofovir gel in preventing HIV acquisition in women. Nat Med. (2018) 24:491–6. 10.1038/nm.4506 29480895 PMC5893390

[55] Sahin-Hodoglugil NN Woods R Pettifor A. Walsh. A Comparison of Cost-Effectiveness of Three Protocols for Diagnosis and Treatment of Gonococcal and Chlamydial Infections in Women in Africa. Sex Transm Dis. (2003) 30:455–69. 10.1097/00007435-200305000-00014 12916139

[56] Reddy SP, James, S, Sewpaul, R, Sifunda, S, Ellahebokus, A, Kambaran, NS, . Umthente Uhlaba Usamila - The 3rd South African National Survey, Youth Risk Behaviour 2011. Cape Town: South African Medical Research Council (2011). p. 4–160. Available from: https://africacheck.org/wp-content/uploads/2018/10/3rd-Annual-Youth-Risk-Survey-2011.pdf

[57] Low N McCarthy A Macleod J Salisbury C Campbell R Roberts TE. Epidemiological, social, diagnostic and economic evaluation of population screening for genital chlamydial infection. Health Technol Assess. (2007) 11:iii–iv, ix–xii, 1–165. 10.3310/hta11080 17311735

[58] Adams EJ Turner KME Edmunds WJ. The cost effectiveness of opportunistic chlamydia screening in England. Sex Transm Infect. (2007) 83:267–74. 10.1136/sti.2006.024364 17475686 PMC2598679

[59] Woods B Revill P Sculpher M Claxton K. Country-level cost-effectiveness thresholds: initial estimates and the need for further research. Value Health. (2016) 19:929–35. 10.1016/j.jval.2016.02.017 27987642 PMC5193154

[60] Meyer-Rath G van Rensburg C Larson B Jamieson L Rosen S. Revealed willingness-to-pay versus standard cost-effectiveness thresholds: Evidence from the South African HIV Investment Case. PLoS ONE. (2017) 12:1–9. 10.1371/journal.pone.0186496 29073167 PMC5658054

[61] Edoka IP Stacey NK. Estimating a cost-effectiveness threshold for health care decision-making in South Africa. Health Policy Plan. (2020) 35:546–55. 10.1093/heapol/czz152 32125375 PMC7225568

[62] Passmore JAS Jaspan HB Masson L. Genital inflammation, immune activation and risk of sexual HIV acquisition. Curr Opin HIV AIDS. (2016) 11:156–62. 10.1097/COH.0000000000000232 26628324 PMC6194860

[63] Lennard K Dabee S Barnabas SL Havyarimana E Blakney A Jaumdally SZ. Microbial composition predicts genital tract inflammation and persistent bacterial vaginosis in South African adolescent females. Infect Immun. (2018) 86:1–18. 10.1128/IAI.00410-17 29038128 PMC5736802

