# Supplementary material for: Cost-effectiveness analysis of different screening and diagnostic strategies for sexually transmitted infections and bacterial vaginosis in women attending primary health care facilities in Cape Town
Source: Front Public Health. 2023 Mar 2;11:1048091. doi: 10.3389/fpubh.2023.1048091 (PMC10018124; doi:10.3389/fpubh.2023.1048091)
Supplement: Supplementary file 1 [file Data_Sheet_1.docx]

Supplementary Material

Appendices


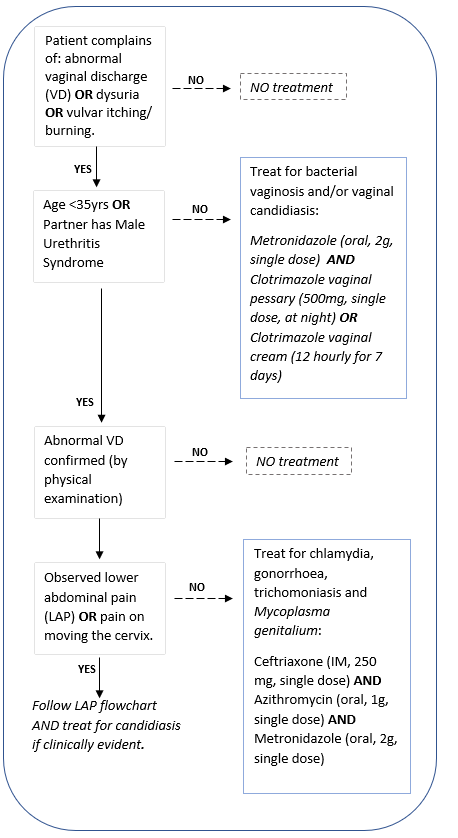
A.1. South African Vaginal Discharge Syndrome flowchart*

Source: Adapted from (1)

*Based on WHO recommendations, but modified for country-specific use

A.2. Decision tree


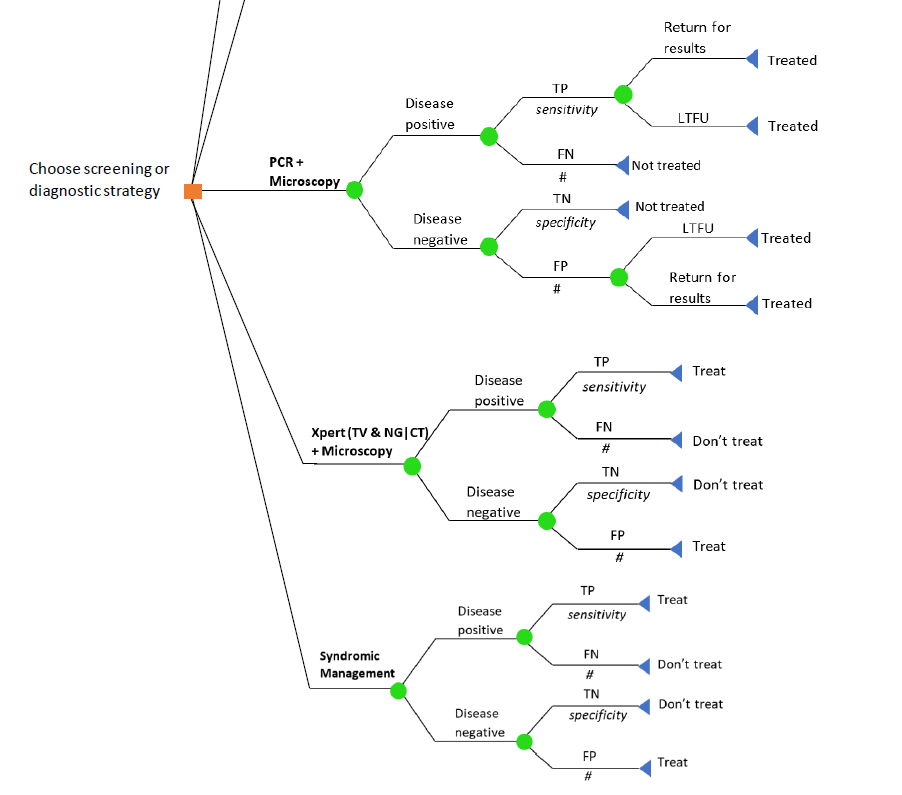

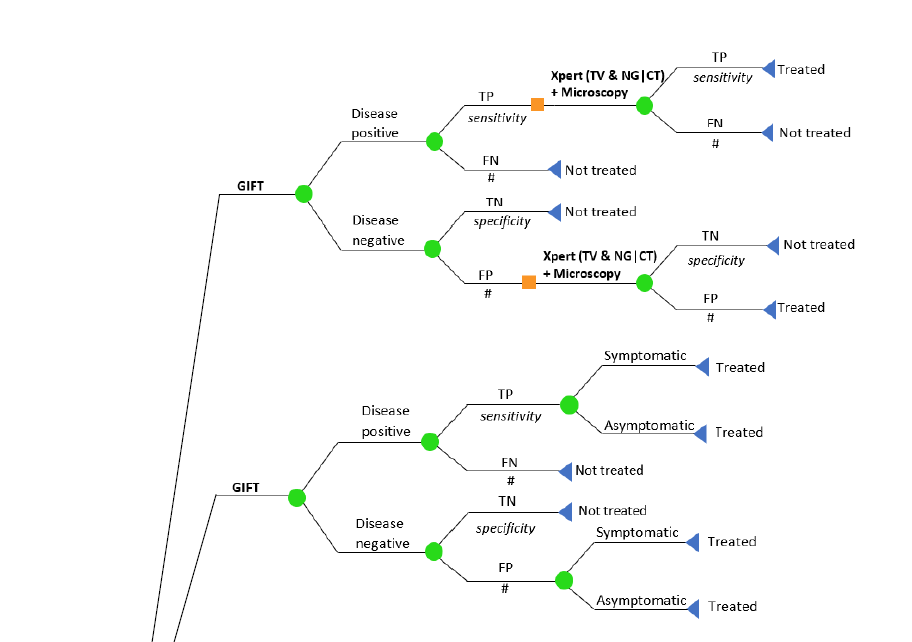


A.3. Model parameters: individual decision analysis

**Bacterial Vaginosis**

| **Parameter** | **Value** | **Source** |
| --- | --- | --- |
| Disease prevalence | 44.5% | (2–13) |
| Syndromic management |  |  |
| Sensitivity | 46.0% | (14) |
| Specificity | 61.6% | (14) |
| GIFT |  |  |
| Sensitivity | 77.0% | (15) |
| Specificity | 71.0% | (15) |
| Microscopy* |  |  |
| Sensitivity | 100% | (Nigel Garret)^[[1]](#footnote-1)^a |
| Specificity | 100% | (Nigel Garret)^[[2]](#footnote-2)^b |

**On-site clinic based and off-site laboratory-based*

***C. trachomatis***

| **Parameter** | **Value** | **Source** |
| --- | --- | --- |
| Disease prevalence | 15.4% | (2–13) |
| Syndromic management |  |  |
| Sensitivity | 44.7% | (14) |
| Specificity | 58.3% | (14) |
| GIFT |  |  |
| Sensitivity | 77.0% | (15) |
| Specificity | 71.0% | (15) |
| GeneXpert NG/CT |  |  |
| Sensitivity | 100% | (16) |
| Specificity | 97.6% | (16) |
| PCR |  |  |
| Sensitivity | 100% | (17) |
| Specificity | 100% | (17) |

***N. gonorrhoeae***

| **Parameter** | **Value** | **Source** |
| --- | --- | --- |
| Disease prevalence | 5.9% | (2–13) |
| Syndromic management |  |  |
| Sensitivity | 45.2% | (14) |
| Specificity | 66.0% | (14) |
| GIFT |  |  |
| Sensitivity | 77.0% | (15) |
| Specificity | 71.0% | (15) |
| GeneXpert NG/CT |  |  |
| Sensitivity | 100% | (16) |
| Specificity | 100% | (16) |
| PCR |  |  |
| Sensitivity | 100% | (17) |
| Specificity | 100% | (17) |

***T. vaginalis***

| **Parameter** | **Value** | **Source** |
| --- | --- | --- |
| Disease prevalence | 10.8% | (2–13) |
| Syndromic management |  |  |
| Sensitivity | 45.6% | (14) |
| Specificity | 60.4% | (14) |
| GIFT |  |  |
| Sensitivity | 77.0% | (15) |
| Specificity | 71.0% | (15) |
| GeneXpert TV |  |  |
| Sensitivity | 96.4% | (18) |
| Specificity | 99.6% | (18) |
| PCR |  |  |
| Sensitivity | 100% | (17) |
| Specificity | 100% | (17) |

References

1. Department of Health. Sexually Transmitted Infections MANAGEMENT GUIDELINES 2015. 2015.

2. van der Eem L, Dubbink JH, Struthers HE, McIntyre JA, Ouburg S, Morré SA, et al. Evaluation of syndromic management guidelines for treatment of sexually transmitted infections in South African women. Tropical Medicine and International Health. 2016;21(9):1138–46.

3. Johnson LF, Dorrington RE, Bradshaw D, Coetzee DJ. The effect of syndromic management interventions on the prevalence of sexually transmitted infections in South Africa. Sexual and Reproductive Healthcare [Internet]. 2011;2(1):13–20. Available from: http://dx.doi.org/10.1016/j.srhc.2010.08.006

4. Kaida A, Dietrich JJ, Laher F, Beksinska M, Jaggernath M, Bardsley M, et al. A high burden of asymptomatic genital tract infections undermines the syndromic management approach among adolescents and young adults in South Africa: Implications for HIV prevention efforts. BMC Infect Dis [Internet]. 2018;18(1):1–11. Available from: https://www.ncbi.nlm.nih.gov/pubmed/30285705

5. Francis SC, Ao TT, Vanobberghen FM, Chilongani J, Hashim R, Andreasen A, et al. Epidemiology of curable sexually transmitted infections among women at increased risk for HIV in Northwestern Tanzania: Inadequacy of syndromic management. PLoS One. 2014;9(7).

6. Barnabas SL, Dabee S, Passmore JAS, Jaspan HB, Lewis DA, Jaumdally SZ, et al. Converging epidemics of sexually transmitted infections and bacterial vaginosis in southern African female adolescents at risk of HIV. Int J STD AIDS. 2018;29(6):531–9.

7. Mlisana K, Naicker N, Werner L, Roberts L, van Loggerenberg F, Baxter C, et al. Symptomatic vaginal discharge is a poor predictor of sexually transmitted infections and genital tract inflammation in high-risk women in South Africa. Journal of Infectious Diseases. 2012;206(1):6–14.

8. Torrone EA, Morrison CS, Chen PL, Kwok C, Francis SC, Hayes RJ, et al. Prevalence of sexually transmitted infections and bacterial vaginosis among women in sub-Saharan Africa: An individual participant data meta-analysis of 18 HIV prevention studies [Internet]. Vol. 15, PLOS Medicine. 2018. 1–38 p. Available from: http://dx.plos.org/10.1371/journal.pmed.1002511

9. Giuliano AR, Botha MH, Zeier M, Abrahamsen ME, Glashoff RH, van der Laan LE, et al. High HIV, HPV, and STI prevalence among young Western Cape, South African women: EVRI HIV prevention preparedness trial. J Acquir Immune Defic Syndr (1988). 2015;68(2):227–35.

10. Garrett NJ, Osman F, Maharaj B, Naicker N, Gibbs A, Norman E, et al. Beyond syndromic management: Opportunities for diagnosis-based treatment of sexually transmitted infections in low- and middle-income countries. PLoS One [Internet]. 2018;13(4):e0196209. Available from: http://journals.plos.org/plosone/article/file?id=10.1371/journal.pone.0196209&type=printable%0Ahttp://ovidsp.ovid.com/ovidweb.cgi?T=JS&PAGE=reference&D=emexb&NEWS=N&AN=621788390

11. Kularatne RS, Niit R, Rowley J, Kufa-Chakezha T, Peters RPH, Taylor MM, et al. Adult gonorrhea, chlamydia and syphilis prevalence, incidence, treatment and syndromic case reporting in South Africa: Estimates using the Spectrum-STI model, 1990-2017. PLoS One [Internet]. 2018;13(10):1–22. Available from: https://journals.plos.org/plosone/article?id=10.1371/journal.pone.0205863

12. Abbai NS, Wand H, Ramjee G. Sexually Transmitted Infections in Women Participating in a Biomedical Intervention Trial in Durban: Prevalence, Coinfections, and Risk Factors. J Sex Transm Dis. 2013;2013:1–6.

13. de Waaij DJ, Dubbink JH, Ouburg S, Peters RPH, Morré SA. Prevalence of Trichomonas vaginalis infection and protozoan load in South African women: A cross-sectional study. BMJ Open. 2017;7(10):1–6.

14. Verwijs MC, Agaba SK, Sumanyi J claude, Umulisa MM, Mwambarangwe L, Musengamana V, et al. Targeted point-of-care testing compared with syndromic management of urogenital infections in women ( WISH ): a cross-sectional screening and diagnostic accuracy study. Lancet Infect Dis. 2019;19:658–69.

15. Masson L, Arnold KB, Little F, Mlisana K, Lewis DA, Mkhize N, et al. Inflammatory cytokine biomarkers to identify women with asymptomatic sexually transmitted infections and bacterial vaginosis who are at high risk of HIV infection. Sex Transm Infect. 2015;92(3):186–93.

16. Garrett N, Mitchev N, Osman F, Naidoo J, Dorward J, Singh R, et al. Diagnostic accuracy of the Xpert CT/NG and OSOM Trichomonas Rapid assays for point-of-care STI testing among young women in South Africa: A cross-sectional study. BMJ Open. 2019;9(2):1–5.

17. Unemo M, Ballard R, Ison C, Lewis D, Ndowa F, Rosanna P. Laboratory diagnosis of sexually transmitted infections, including human immunodeficiency virus. World Health Organization [Internet]. 2013;244. Available from: http://www.fidssa.co.za/images/LR_WHO_lab_manual_2013.pdf

18. Gaydos CA, Klausner JD, Pai NP, Kelly H, Coltart C, Peeling RW, et al. Vaginalis in Women and Men. 2017;93(Suppl 4).

1. a Personal communication: Nigel Garret, 29 May 2019 [↑](#footnote-ref-1)
2. b Same as above [↑](#footnote-ref-2)
